# Pneumonia

**Published:** 1898-12

**Authors:** R. W. Wallis

**Affiliations:** Rockdale Texas


For Texas Medical Journal.

### Pneumonia.

BY R. W. WALLIS, M. D., ROCKDALE TEXAS.

Read before the Brazos Valley Medical Association, at its sixth semi-annual meeting at Rockdale, Texas, Nov. 15 and 16, 1898.

Pneumonia is one of the oldest diseases known to medicine, and one in which no difference of opinion prevails in regard to its pathology. Years ago it was looked upon as being one of the most typical of inflammatory diseases and the most active anti-phlogistic treatment was adopted to overcome it. Venesection was practiced to the point of syncope, while calomel and tartar-emetic were administered internally and blisters were applied externally. It infects all ages, especially early and late periods of life, is an infectious self-limited disease, and it is questionable whether it ever arises from cold, but due to a diplococcus pneumoniæ; but this is not a characteristic of the disease as this germ is also found in bronchitis. Banti gives us as the most notable feature of his research "The almost invariable presence of the diplococcus lanceolatus in lobar pneumonia, not only in the exudation, the lung and pleura, but often in the blood, and that variations in the intensity of the disease seems to depend on differences in the virulence of the microbe, complications as a rule being excited by the same agency." Bozzola cites quite an interesting case in which the diplococcus of pneumonia occurred in the milk of a woman affected with pneumônia. "The patient, who was nursing a child five months old, was attacked by a pneumonia crouposa sinistra which later on extended to the right lung and was followed by endocarditis and ended in lysis; cultures of milk pressed from the breasts on the fifth day of the disease gave rich development of pneumococci." Immunity against pneumonia pneumococcus by experiments on rabbits, has revealed that every nutrient medium in which the pneumococcus has been cultivated, will if inoculated, render an animal immune against pneumonic septicemia, even after the cocci have been removed by filtration. It is claimed that an outbreak of pneumonia may be prevented by protective injections, while if the disease is once established it may be cured by the same means; the immunity or cure is to be obtained by injections of blood or juice from the tissues of immunized animals. It has been demonstrated that the pneumococcus, when introduced into the body of an animal generates a poisonous substance which can be isolated and which has received the name pneumotoxin; this substance sets up a febrile condition which lasts several days, after which another substance is found to have been produced called anti-pneumotoxin, which is the substance that cures an attack of pneumonic-septicemia in other animals. It was demonstrated by Klemperer that the serum taken from the pneumonic patients after the crisis could cure pneumonia in rabbits, the human body being much less susceptible than rabbits; moreover pneumotoxines were found to be present in human serum and in that taken from rabbits.

According to various authors the crisis of pneumonia takes place as soon as anti-pneumotoxin is produced in sufficient quantities to neutralize the pneumotoxine. The prognosis is generally good in the young, but grave in old age; danger increases as to the amount of lung substance involved. It would no doubt, be a great gain if we could recognize approaching dangerous crises and thus diminish the number of so-called sudden and unexpected deaths. In the weak, elderly, and drunkard the prognosis must be made with extreme care, for no general rule founded on shattered constitutions will apply. There are a number of bad symptoms, so to speak, which it might be well to mention, and when they occur it is always well to note them with suspicion, such as dullness on percussion, with tubular breathing when it occurs early in the disease; a total absence of expectoration is very rare and significent, and on the other hand a large quantity of frothy bronchial secretion, a continued high temperature of 104 F. from the first, and if it reaches 105 F. on two consecutive days without any material decline it is very dangerous; the outlook is always serious in adults, when the acceleration in respiration has maintained a rate of about 50 per minute; about the sixth or ninth day a constantly extending area of dullness is very unfavorable; also, a steadily increasing rate of pulse at this time must be watched. Delirium in any condition is dangerous; as it has been so ably expressed as "the idle comments of the brain that foretell the ending of man," it must be especially watched if it occurs early in the attack. But the most interesting part of this subject to us is the treatment; suffice it to say that eighty-five out of every hundred cases would get well without any treatment, as we remember it is a self-limited disease and a patient can be easily killed by too much treatment; as there are no specifics; we treat symptoms and not the disease. So when most of the essential points in the treatment are summed up, it consists simply to avoid cardiac sedatives, give good nutriment and hygiene, and let the patient get well. I have little or no confidence in the so-called cold treatment as used by the Germans, for I do not believe that it is merely a local trouble in the lung which is wholly responsible for the elevated temperature, but that the disease is a constitutional one and its chief danger arises from the high fever, which finally leads to cardiac failure, so the principal aim is to reduce the fever at large and give supportive treatment.

As soon as the diagnosis is made, you must support the heart to help overcome the obstruction; if the pulse is good, commence with a minimum amount of stimulation, depending of course upon the patient.

Alcoholics generally prove fatal, as they are habituated to stimulants, therefore have least resistance; the pulse being of lowered tension and rapid, must be watched, for these cases die suddenly; and it matters not how little of lung tissue is involved, never give a good prognosis, especially if the patient has age against him, so give enough of stimulants to keep pulse steady; and here is where whisked, brandy, etc., although I dislike to advocate it, finds a valuable nlace in medicine; it has to be given according to the patient. If the heart begins to flag, give digitalis, a fresh infusion of the leaves or a reliable tincture. There is an objection to digitalis, however, as it makes the pulse wiry, so the best results are obtained by changing to strophanthis and vice versa. Digitalis should always be watched with care, for it sometimes tetanizes the heart; give from five to fifteen drops. Ammonia is given to liquify the fibro-plastic material, as it is a good diffusible stimulant. I prefer the muriate, giving five or ten grains every four hours. This is not given as an expectorant, for it is very nauseating and would weaken the patient if given that way; also we must sustain the stomach. I take it that expectorants in the early stages of pneumonia are not worth the time it takes to mention it, as they can onlv be useful in the third stage. Pain is one symptom that doctors are first called upon to relieve, and I consider a physician's first mission, that which places him in a true light, is to be able to give such remedies as will relieve pain, to produce sleep,

"Sleep that knits up the ravelled sleeve of care,
The death of each day's life; sore labour's bath,
Balm of hurt minds, great Nature's second course,
Chief nourisher in life's feast."

As a pneumonic patient must sleep, give sulphonal ten to twenty grains at bed-time, with half a glass of warm milk. The pain must be relieved, so give opium hypodermically; morphine has a tendency to deaden the respiratory center, so commence with small doses, as one-tenth or one-twelfth of a grain.

Codia sometimes acts very well; poultices would be good, but they have to be changed too often, therefore nothing is superior to cotton and oil silk; envelope the chest from the clavicle to the lower ribs, with the cotton bound tolerably tight. Look after the secretions; it is not necessary for the bowels to act every day, but every two or three days. Small doses of calomel and soda or the elixir of Cascara Sagrada acts quite well. Give the patient all nourishment possible, as milk, beef juice, etc., and especially milk punch, which give from the first. It is well to remember that anti-pyretics are cardiac depressants, so give as few as possible; phenacetine I consider the best; commence with small doses, three to five grains every hour, and relieve the cephalalgia. Of course quinine is to be given, and should be given first, last and all the time. I prefer it in large doses as far apart as possible, and give plenty of water. The lancet, although relegated to the past, I think is too little used; I fear we are ruled too much by our prejudices. I think there are a number of cases, plethoric, robust individuals, who, if the lancet had been used early, would have had little trouble. Strychnia has a very important place in the treatment of pneumonia, especially when the pulse is in a depressed stage: then it must be given full and free, as one-thirtieth of a grain every three or four hours; there appears to be a wonderful tolerance of this drug by pneumonic patients, but the pulse must be watched. In chronic unresolved cases where after six or ten weeks there has been no absorption, blisters and the iodides of pot. and amm. have an excellent place. I take it that purgatives are never needed; laxatives, however, are good and are often made use of.

When symptoms of cyanosis or coma manifest themselves, oxygen must be given without stint by inhalations.

All that the term good nursing in its broadest sense implies, aids materially in producing a rapid convalescence.

